# P-976. Impact of an Antimicrobial Stewardship Rotation for Internal Medicine Residents

**DOI:** 10.1093/ofid/ofaf695.1175

**Published:** 2026-01-11

**Authors:** Marcela Araujo de Oliveira Santana, Noah Romero Nakajima, Darcy Wooten, Sena Sayood

**Affiliations:** Washington University in St Louis, St Louis, MO; Washington University in St Louis, St Louis, MO; Washington University in St. Louis, Kirkwood, MO; Washington University School of Medicine, St. Louis, Missouri

## Abstract

**Background:**

Antimicrobial stewardship (AS) is critical to addressing rising antimicrobial resistance. Internal Medicine (IM) residents are a key audience for targeted education, yet most existing curricula emphasize knowledge acquisition outside the clinical context. To address this gap, we developed a clinically embedded AS rotation to enhance residents’ applied knowledge, attitudes, and confidence in antimicrobial use.

**Table 1:**
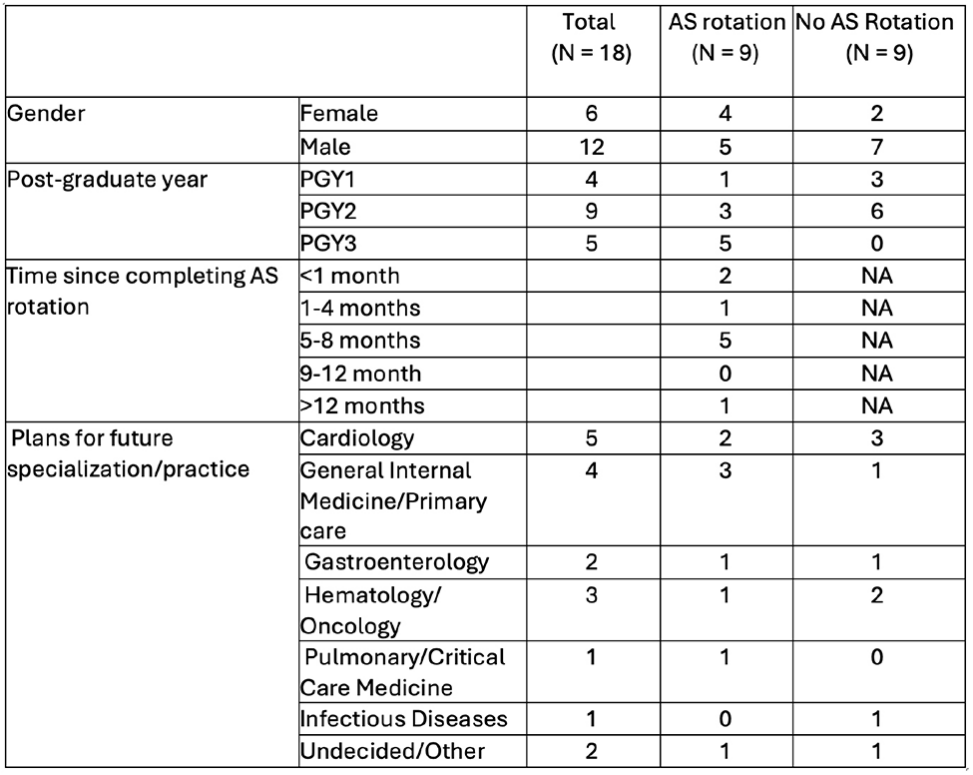
Characteristics of Internal Medicine Residents, Stratified by Participation in the Antimicrobial Stewardship (AS) Rotation

**Figure 1:**
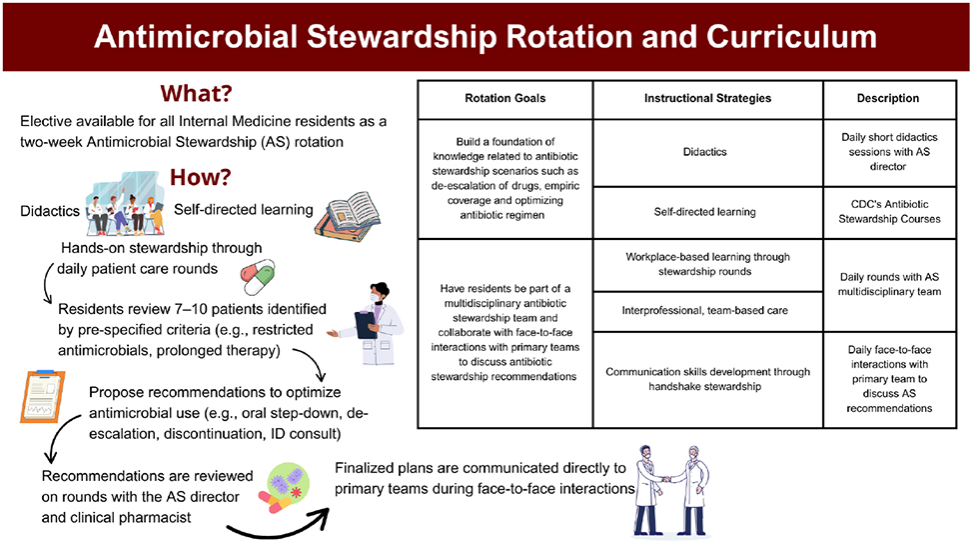
Overview of the Antimicrobial Stewardship (AS) rotation, goals, and instructional strategies.

**Methods:**

Using the Kern Model and grounded in Constructivist learning theory, we designed a 2-week AS rotation and curriculum for IM residents (Figure 1). Educational outcomes were evaluated via an anonymous survey (Qualtrics) assessing AS knowledge application, attitudes, and self-perceived skills. All Washington University/BJC IM residents (N=160) during the 2024–2025 academic year were eligible. Residents who completed the rotation before April 1, 2025, formed the intervention group; those who had not participated in the rotation served as controls. Group comparisons used chi-square or Fisher’s exact tests for categorical variables and independent t-tests or Mann-Whitney U tests for continuous variables. A p-value < 0.05 was considered significant. The study was IRB-exempt.

**Figure 2:**
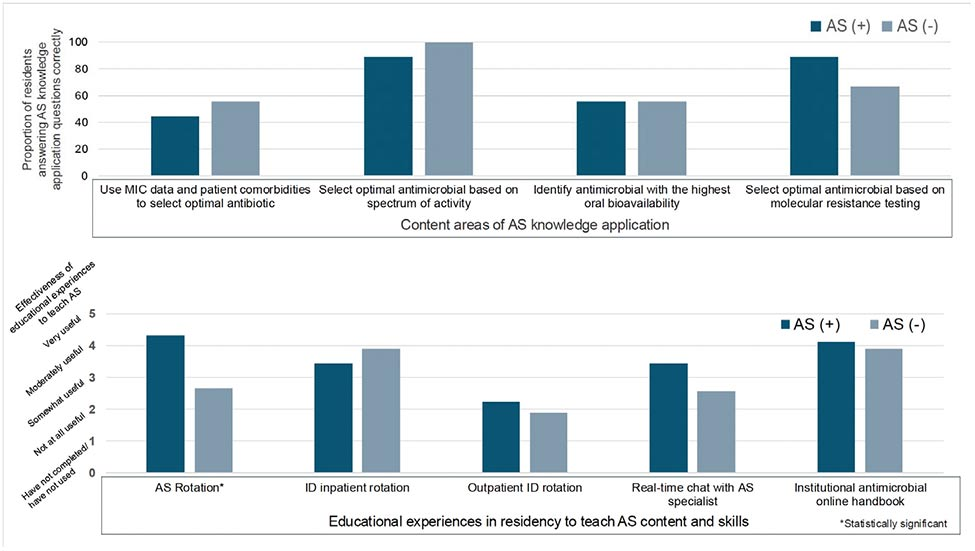
Antimicrobial Stewardship (AS) Knowledge Application Outcomes (N=18). There were no statistically significant differences in AS knowledge application between residents who participated in (AS+) and did not participate in (AS-) the antimicrobial stewardship rotation. Residents’ self-report of the effectiveness of various educational experiences in residency to teach AS content and skills (N = 18). Residents who completed the AS rotation found it to be the most useful instructional strategy to teach AS content and skills and they found the AS rotation to be significantly more useful than the residents who did not complete the rotation (P<0.05).

**Figure 3:**
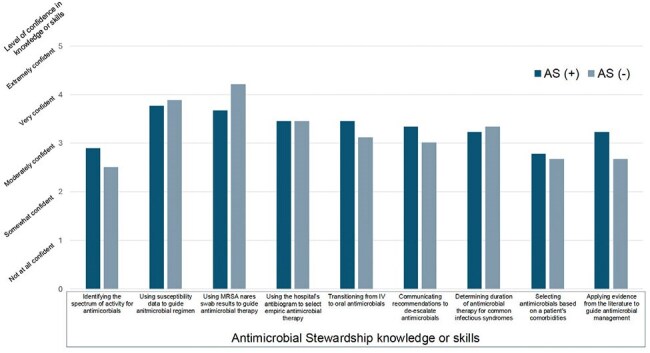
Residents’ self-report of confidence in various AS skills (N = 18). There was no statistically significant difference between residents who had completed the AS rotation and those who did not.

**Results:**

In this interim analysis, 18 residents (11%) responded to the survey; 9 had completed the AS rotation (Table 1). No statistically significant differences were observed in AS knowledge application (Figure 2). However, rotation participants reported greater confidence in stewardship knowledge and skills and more frequent use of AS tools (e.g., antibiograms), though these differences were not statistically significant (Figure 3). The AS rotation was rated as the most effective instructional strategy for improving antimicrobial decision-making (Figure 2).

**Conclusion:**

A structured AS rotation offers meaningful, hands-on training for IM residents. While early findings are limited by sample size, trends suggest improved confidence and engagement with AS principles. Ongoing data collection is underway to assess impact and guide future curricular development.

**Disclosures:**

All Authors: No reported disclosures

